# Deep Reinforcemnet Learning for Robust Beamforming in Integrated Sensing, Communication and Power Transmission Systems

**DOI:** 10.3390/s25020388

**Published:** 2025-01-10

**Authors:** Chenfei Xie, Yue Xiu, Songjie Yang, Qilong Miao, Lu Chen, Yong Gao, Zhongpei Zhang

**Affiliations:** 1 National Key Laboratory of Science and Technology on Communications, University of Electronic Science and Technology of China, Chengdu 611731, China; chenff93625@163.com (C.X.); xiuyue12345678@163.com (Y.X.); yangsongjie@std.uestc.edu.cn (S.Y.); 2 School of Information and Communication Engineering, University of Electronic Science and Technology of China, Chengdu 611731, China; 201911012021@std.uestc.edu.cn; 3 School of Aeronautics and Astronautics, University of Electronic Science and Technology of China, Chengdu 611731, China; lchen@std.uestc.edu.cn; 4 School of Computer Science and Information Engineering, Hefei University of Technology, Hefei 230601, China; gy_eee@163.com

**Keywords:** integrating sensing, communication, power transfer, deep reinforcement learning, robust beamforming, imperfect channel state information, multi-user

## Abstract

A communication network integrating multiple modes can effectively support the sustainable development of next-generation wireless communications. Integrated sensing, communication, and power transfer (ISCPT) represents an emerging technological paradigm that not only facilitates information transmission but also enables environmental sensing and wireless power transfer. To achieve optimal beamforming in transmission, it is crucial to satisfy multiple constraints, including quality of service (QoS), radar sensing accuracy, and power transfer efficiency, while ensuring fundamental system performance. The presence of multiple parametric constraints makes the problem a non-convex optimization challenge, underscoring the need for a solution that balances low computational complexity with high precision. Additionally, the accuracy of channel state information (CSI) is pivotal in determining the achievable rate, as imperfect or incomplete CSI can significantly degrade system performance and beamforming efficiency. Deep reinforcement learning (DRL), a machine learning technique where an agent learns by interacting with its environment, offers a promising approach that can dynamically optimize system performance through adaptive decision-making strategies. In this paper, we propose a DRL-based ISCPT framework, which effectively manages complex environmental states and continuously adjusts variables related to sensing, communication, and energy harvesting to enhance overall system efficiency and reliability. The achievable rate upper bound can be inferred through robust, learnable beamforming in the ISCPT system. Our results demonstrate that DRL-based algorithms significantly improve resource allocation, power management, and information transmission, particularly in dynamic and uncertain environments with imperfect CSI.

## 1. Introduction

As wireless networks evolve to meet the increasing demands of ultra-reliable low-latency communication (uRLLC), massive machine-type communication (mMTC), and enhanced mobile broadband (eMBB), the efficient utilization of available spectrum and the improvement of signal quality have become critical priorities [1,2,3]. In this context, integrated sensing and communications (ISAC) has emerged as a promising solution, enabling the simultaneous transmission of data and environmental sensing via shared wireless signals [4,5]. Concurrently, the development of energy-harvesting technologies has generated interest in simultaneous wireless information and power transfer (SWIPT), which extends the operational lifespan of wireless devices while maintaining high-quality data transmission [6]. SWIPT has shown significant promise in applications such as the Internet of Things (IoT) and low-power device scenarios [7,8,9]. Building on these advancements, the concept of integrated sensing, communications, and power transfer (ISCPT) has been introduced. ISCPT combines the principles of ISAC and SWIPT to create a versatile system that not only transmits information and harvests energy, but also gathers situational awareness within a unified framework [10]. Using shared signal designs and optimized resource allocation, ISCPT systems have the potential to transform traditional wireless networks by simultaneously addressing the challenges of data transmission, environmental sensing, and energy harvesting [11,12].

The principles underpinning ISCPT highlight the complementary nature of its constituent technologies [13]. ISCPT systems have demonstrated substantial capacity in improving spectrum efficiency, energy sustainability, and system scalability. A core area of investigation in ISCPT research is signal design and resource optimization, where researchers aim to develop multi-functional waveforms that address diverse system requirements [14,15]. Furthermore, advances in hardware architecture, including energy-harvesting circuits and sensing-optimized antenna designs, have enabled the practical deployment of ISCPT systems [16]. Among these enabling technologies, beamforming is a fundamental technique in ISCPT systems, as it governs the spatial allocation of wireless signals to optimize overall system performance [17,18,19,20,21,22]. Traditional beamforming approaches often rely on static optimization frameworks that assume precise and accurate knowledge of the environment and system parameters. For instance, in [23], a joint optimization problem involving power allocation and beamforming in wideband millimeter-wave (mmWave) channels is proposed, leveraging limited feedback on channel information. However, in practical deployments, obtaining perfect or complete channel state information (CSI) is often challenging due to various factors such as hardware limitations, feedback delays, and environmental dynamics. Imperfect CSI introduces uncertainties that can significantly degrade the performance of ISCPT systems. It affects critical aspects such as beamforming accuracy, power transfer efficiency, and sensing reliability, leading to suboptimal system operation. For example, inaccurate CSI may result in incorrect spatial signal allocation, increasing interference and reducing the achievable communication rate. Moreover, imperfect CSI complicates the optimization problem by introducing non-convexity and stochastic variations, making traditional static approaches less effective [24]. Addressing these challenges requires adaptive and robust optimization methods capable of dynamically handling uncertainties in CSI while maintaining high system performance.

Recent advancements in artificial intelligence (AI) and optimization techniques have provided innovative solutions for dynamically allocating resources [25] and mitigating interference [26]. To ensure robust system performance across heterogeneous network environments, deep learning (DL) has emerged as a particularly promising approach for physical-layer optimization. DL offers data-driven methods to optimize beamforming without relying on exact system models or perfect environmental knowledge. Moreover, deep reinforcement learning (DRL) introduces a novel paradigm for beamforming by modeling the optimization problem as a Markov decision process (MDP) [27,28,29,30,31]. In this framework, an agent dynamically selects beamforming actions based on environmental states. DRL leverages neural networks to approximate policies or value functions, enabling efficient exploration and exploitation in non-convex and stochastic environments, such as those encountered in reconfigurable intelligent surfaces (RISs) [32], cell-free networks [33], and movable antennas [34]. By enabling multiple functionalities with wireless communication and signal processing, DRL significantly enhances spectrum utilization, addressing the challenges posed by limited bandwidth in densely populated networks [35]. One of the most compelling advantages of DRL is its ability to maintain strong robustness in the presence of environmental uncertainties, dynamic conditions, and imperfect CSI. Unlike traditional optimization methods, which rely on fixed assumptions about system parameters and often suffer from performance degradation under mismatched conditions, DRL dynamically adapts to varying environments by continuously learning from real-time interactions [36]. This adaptability makes DRL especially effective in scenarios with stochastic dynamics, such as rapid channel fluctuations, unexpected interference, and mobility-induced changes [37]. For example, in ISCPT systems, where multiple objectives, including communication, sensing, and power transfer, compete for resources, DRL agents can adjust their policies in response to real-time feedback, ensuring that the system maintains optimal performance even under adverse conditions. So far, different agents can also design to deal with the relationship between each group of variables according to different state representations. In ISAC system, DRL can collect specialized waveforms that are optimized for both high communication rates and accurate sensing, ensuring minimal trade-offs between the two functions [38]. By allowing devices to harvest wireless energy, SWIPT reduces dependence on traditional power sources, enabling extended operation in battery-constrained scenarios. It enables devices such as IoT sensors and wearable electronics to harvest energy from ambient signals while maintaining seamless communication [39].

This capability makes DRL particularly well suited for ISCPT systems, where beamforming must balance competing objectives—sensing accuracy, communication quality, and harvested energy—while adapting to dynamic network conditions. DRL combines the adaptability of reinforcement learning with the representational power of deep neural networks, enabling the learning of optimal policies in high-dimensional, uncertain environments. DRL excels in environments with stochastic dynamics, making it ideal for ISCPT systems, where channel conditions, interference, and user requirements are constantly evolving. By modeling optimization as an MDP, DRL allows intelligent agents to learn policies that adapt to these changing conditions [32]. ISCPT systems require a delicate balance between sensing, communication, and power transfer objectives, which can be achieved through DRL by utilizing reward functions that navigate the trade-offs among these competing goals. For instance, a DRL agent can dynamically prioritize sensing accuracy in critical scenarios or optimize energy transfer when device power levels are low. DRL’s ability to explore the solution space enables the identification of better-performing policies. Once properly trained, the framework can generalize across diverse network configurations and operational conditions, making it highly scalable for large-scale ISCPT deployments involving multiple users and devices. DRL learns from interactions with the environment, allowing it to handle uncertainties and unpredictable interference patterns. Beamforming is central to ISAC, as it directs signal energy for both sensing and communication [29]. DRL-based methods dynamically optimize beam patterns to guarantee sensing accuracy and communication quality under dynamic conditions. DRL has also been used to optimize resource allocation in SWIPT systems, ensuring efficient energy harvesting while maintaining communication reliability. This is particularly useful in IoT networks, where devices have stringent energy constraints. ISCPT systems involve interactions across multiple layers, including the physical, media access control (MAC), and application layers. DRL enables cross-layer optimization by learning policies that account for dependencies and trade-offs across these layers. DRL-powered ISCPT systems can support applications such as real-time video streaming in autonomous vehicles, where high communication rates and low latency must coexist with environmental sensing and energy harvesting.

Achieving robust beamforming in ISCPT systems is critical for supporting real-world IoT applications. By integrating DRL with ISCPT technologies, this study aims to address the critical challenges in multi-objective optimization and real-time adaptability, providing a foundation for advanced solutions in future wireless networks. This paper investigates the application of DRL to robust beamforming in ISCPT systems, addressing the trade-offs among multi-user communication quality, sensing accuracy, and harvested energy efficiency. Specifically, we propose a DRL-based framework that jointly optimizes beamforming vectors while accounting for system uncertainties and dynamic network conditions. The key contributions of this work are as follows:

- **DRL Framework for ISCPT Beamforming**: We design and implement an innovative DRL-based framework tailored for beamforming in ISCPT systems. By simulating a dynamic environment, the DRL agent learns optimal beamforming policies through iterative trial-and-error interactions. This approach captures complex system behaviors and provides a scalable solution for optimizing multi-objective performance. Unlike traditional optimization methods, the DRL framework enables continuous adaptation to changes in CSI and environmental dynamics, ensuring robust and efficient beamforming under varying conditions.
- **Achievable Upper Bound in ISCPT System**: To quantify and maximize system performance, we formulate a joint optimization problem aimed at achieving the theoretical upper bound of ISCPT systems. This involves simultaneous optimization of transmit beamforming vectors while adhering to stringent system constraints, such as power budgets, quality-of-service requirements, and energy-harvesting thresholds. Furthermore, the framework addresses critical challenges posed by uncertainties in CSI and dynamically evolving parameters, ensuring a balance among communication, sensing, and energy objectives.
- **Robustness to Environmental Dynamics**: The proposed framework demonstrates exceptional adaptability and resilience in realistic scenarios. By leveraging DRL’s capability to learn from dynamic interactions, the system achieves robust performance even under unpredictable environmental conditions. Extensive simulations validate the effectiveness of our approach, showcasing significant improvements in multi-objective performance metrics compared to conventional optimization techniques. This adaptability positions the framework as a practical solution for next-generation wireless networks requiring real-time and robust optimization.

This work bridges the gap between the emerging field of ISCPT and advanced machine learning techniques, offering a scalable and robust solution for beamforming in multi-functional wireless networks. The proposed framework significantly enhances the practicality and efficiency of ISCPT systems, paving the way for their deployment in next-generation communication networks, including 6G and beyond.

This paper is organized as follows: Section 2 introduces the novel integrated IoT devices and sensing target communication model. Section 3 presents the problem formulation and derives the upper bound of the achievable rate under imperfect CSI. Section 4 proposes the DRL framework for joint transmit beamforming design in ISCPT systems. Section 5 provides the corresponding configurations and demonstrates the performance of the proposed algorithm through numerical results. Finally, Section 6 summarizes the findings of this paper and outlines future research directions.

*Notations*: ${\left(\cdot \right)}^{T}$ and ${\left(\cdot \right)}^{H}$ denote transpose and conjugate transpose, respectively. ${∥\cdot ∥}_{2}$ and tr(·) represents Euclidean norm and trace, respectively. ⊙ expresses the Hadamard Product, and C is the sets of complex numbers. Scalar *x* is represented by lowercase letters, vector x by bold lowercase letters and matrice X by bold uppercase letters.

## 2. System Model

**Figure 1:**
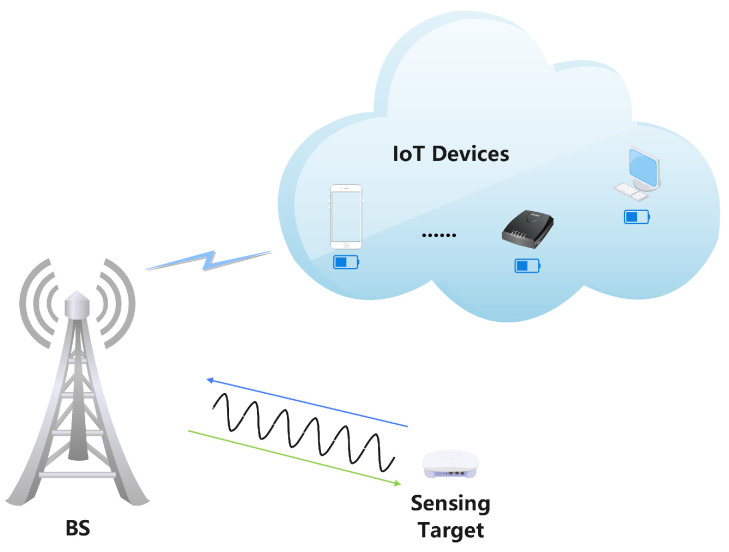
Illustration of the integrated IoT devices and sensing target communication model.

This section presents the system model for the ISCPT system. As depicted in Figure 1, the system consists of a base station (BS) equipped with $N_{t}$ antennas, $K(K<N_{t})$ single-antenna IoT devices, and a target that needs to be perceived. The BS not only facilitates communication with the IoT devices but also transfers energy to sustain their operations. Simultaneously, the BS engages in target sensing by transmitting dedicated signals designed for environmental perception. The integration of communication, power transfer, and sensing is crucial to the system’s functionality and efficiency. In the following subsections, we provide a detailed description of the channel propagation model, data transmission model, energy-harvesting model, and target sensing model that form the foundation of this integrated system.

### 2.1. Wireless Channel Propagation Model

This section clarifies the wireless channel model used for communication between the BS and the IoT devices. The channel between the BS and the *k*-th IoT device, denoted as $h_{k}\in C^{N_{t}\times 1}$, consists of two components: line-of-sight (LOS) $h_{k,\mathrm{LOS}}$ and non-line-of-sight (NLOS) $h_{k,\mathrm{NLOS}}$. The overall channel is the sum of these two components, where the LOS provides a direct path and the NLOS accounts for multipath effects such as reflections, scattering, and diffraction.

The BS is equipped with a uniform linear array (ULA), and the response of the antenna array to a received signal from the *k*-th IoT device depends on the angle of departure (AoD) $\theta_{k}$. The array response can be represented by the array steering vector $a\left(\theta_{k}\right)$, which is a function of the angle of arrival $\theta_{k}$. The steering vector for an antenna array in the far field is given by

(1) $$a\left(\theta_{k}\right)=\frac{1}{\sqrt{N_{t}}}{\left[1,e^{j\frac{2\pi d}{\lambda }\sin\left(\theta_{k}\right)},\ldots ,e^{j\frac{2\pi d}{\lambda }\left(N_{t}-1\right)\sin\left(\theta_{k}\right)}\right]}^{T},$$

where *d* is the distance between adjacent antenna elements, and λ is the carrier wavelength.

The LOS channel component $h_{k,\mathrm{LOS}}$ is deterministic and represents the direct line-of-sight path between the BS and the IoT device. This component is typically modeled as

(2) $$h_{k,\mathrm{LOS}}=\sqrt{\beta_{k,\mathrm{LOS}}}⊙a\left(\theta_{k}\right),$$

where $\beta_{k,\mathrm{LOS}}$ is the path gain of the LOS component, which accounts for free-space path loss and large-scale fading effects. The NLOS $h_{k,\mathrm{NLOS}}$ component accounts for multipath propagation caused by reflections, scattering, and diffractions. The overall channel between the BS and the *k*-th IoT device combines both LOS and NLOS components as follows:

(3) $$h_{k}=h_{k,\mathrm{LOS}}+h_{k,\mathrm{NLOS}}=\sqrt{\frac{\kappa }{\left(1+\kappa \right)}}a\left(\theta_{k}\right)+\frac{1}{\sqrt{1+\kappa }}g_{k},$$

where $\beta_{k,\mathrm{NLOS}}$ is the path gain of the NLOS component, which depends on the scattering environment and large-scale fading. κ denotes the Rician factors, and $g_{k}$ is the small-scale fading vector, representing independent and identically distributed (i.i.d.) complex Gaussian random variables for each antenna.

This composite channel model, combining both the deterministic LOS path and the random NLOS multipath effects, provides a realistic representation of the wireless channel in typical communication environments.

### 2.2. Data Transmission Model

For data transmission, the BS utilizes beamforming to direct the signal toward the IoT devices. The signal transmitted to the *k*-th IoT device is given by $s_{k}\left(t\right)=w_{k}x_{k}\left(t\right)$, where $w_{k}\in C^{N_{t}\times 1}$ is the beamforming vector for the *k*-th device, and $x_{k}\left(t\right)$ is the transmitted signal, with the condition $E\{x_{i}\left(t\right)x_{j}^{H}\left(t\right)\}=1$ for i≠j, ensuring the signals are orthogonal. The received signal at the *k*-th IoT device is influenced by both the desired signal and interference from other devices. The channel is modeled as an additive white Gaussian noise (AWGN) channel, and the received signal at the *k*-th IoT device is

(4) $$y_{k}\left(t\right)=\sqrt{\rho_{k}}\left[h_{k}^{H}s_{k}\left(t\right)+\sum_{j\neq k}h_{k}^{H}s_{j}\left(t\right)+n_{k}\left(t\right)\right]+{\bar{n}}_{k}\left(t\right),$$

where $\rho_{k}\left(0\leq \rho_{k}\leq 1\right)$ is the power split factor between communication and power transfer for the *k*-th device, $n_{k}\left(t\right)∼\mathrm{CN}\left(0,\sigma_{k}^{2}\right)$ and ${\bar{n}}_{k}\left(t\right)∼\mathrm{CN}\left(0,{\bar{\sigma }}_{k}^{2}\right)$ denote the additional complex Gaussian noises. The beamforming vector $w_{k}$ is typically chosen to maximize the received signal power at the *k*-th device, improving the signal-to-interference-plus-noise ratio (SINR). The SINR for the *k*-th device, which is a key metric for evaluating the quality of the received signal, is given by the ratio of the desired signal power to the total interference (from other devices) and noise power. The SINR is formulated as

(5) $${\mathrm{SINR}}_{k}=\frac{\rho_{k}{\left|h_{k}^{H}w_{k}\right|}^{2}}{\rho_{k}\sum_{j\neq k}{\left|h_{k}^{H}w_{j}\right|}^{2}+\rho_{k}\sigma_{k}^{2}+{\bar{\sigma }}_{k}^{2}}.$$

### 2.3. Energy-Harvesting Model

In the proposed system, the BS not only communicates with the IoT devices but also transfers power to sustain their operations using SWIPT. The power transfer is performed concurrently with data communication by combining the communication signal with the power transfer signal. The received signal for power harvesting at the *k*-th IoT device is given by

(6) $$y_{k}^{EH}\left(t\right)=\sqrt{1-\rho_{k}}\left[h_{k}^{H}s_{k}\left(t\right)+\sum_{j\neq k}^{K}h_{k}^{H}s_{j}\left(t\right)+n_{k}\left(t\right)\right].$$

The energy-harvesting process depends on the signal strength and the efficiency of the energy conversion. To reduce interference and simplify the signal processing, the transmitted signal for the *k*-th device satisfies $E\left[{\left|x_{k}\right|}^{2}\right]=1$. The energy collected by the *k*-th IoT device can be followed as

(7) $$E_{k}=\eta_{k}\left(1-\rho_{k}\right)\left[{\left|h_{k}^{H}w_{k}\right|}^{2}+\sum_{j\neq k}^{K}{\left|h_{k}^{H}w_{j}\right|}^{2}+\sigma_{k}^{2}\right],$$

where $\eta_{k}\in \left(0,1\right]$ is the energy conversion efficiency for the *k*-th IoT device. This model assumes a linear energy conversion process and no interference between the information and power transfer signals. The model presented here captures the dual-functionality of the BS in simultaneously serving communication and power transfer needs for the IoT devices, which is critical for the operation of energy-constrained devices in the system.

### 2.4. Sensing Signal Model

Along with communication and energy harvesting, the BS is responsible for sensing a target in the environment. The BS directs beams towards the target and analyzes the signals that are reflected or scattered by the target to estimate its position and properties. For simplicity, the target is assumed to only reflect the transmitted signals, and the channel $h_{s}\in C^{N_{t}\times 1}$ is considered with a LOS path similar as (2). The received signal at the BS is then used for detection and localization [5]. The received signal from the target at the BS can be expressed as

(8) $$y_{s}\left[n\right]=\sum_{i=1}^{K}\alpha h_{s}h_{s}^{H}w_{i}x_{i}\left[n\right]+n_{s},$$

where α is the reflection coefficient of the target, $h_{s}$ is the channel vector between the BS and the target, and $n_{s}$ represents the noise associated with the target sensing. The BS employs beamforming to enhance the signal reflected by the target. The channel between the BS and the target can be represented by the steering vector ${\bar{a}}_{T}\left(\varphi_{DoA}\right)\in C^{N_{Tx}\times 1}$, which depends on the direction of arrival (doa) $\varphi_{DoA}$ of the reflected signal. The steering vector is defined as

(9) $${\bar{a}}_{T}\left(\varphi_{DoA}\right)={\left[1,e^{-j\pi \sin\varphi_{DoA}},\ldots ,e^{-j\left(N_{t}-1\right)\pi \sin\varphi_{DoA}}\right]}^{T}.$$

The target detection is enhanced by time-of-flight (ToF) measurements, which help improve the accuracy of the target’s localization [40]. The total received signal over *N* sampling moments is

(10) $$Y_{s}=\left[\begin{array}{c} y_{s}\left[1\right]\\ \vdots \\ y_{s}\left[N\right] \end{array}\right]=\left[\begin{array}{c} \sum_{i=1}^{K}\alpha h_{s}h_{s}^{H}w_{i}x_{i}\left[1\right]+n_{s}\left[1\right]\\ \vdots \\ \sum_{i=1}^{K}\alpha h_{s}h_{s}^{H}w_{i}x_{i}\left[N\right]+n_{s}\left[N\right] \end{array}\right],$$

where $Y_{s}$ is received signal corresponding to the target. To simplify the expression, it is intuitional that can define $H_{s}={\left(h_{s}^{H}\right)}^{H}h_{s}^{H}\in C^{N_{t}\times N_{t}}$ and $S=\left[w_{i}x_{i}\left[1\right],\ldots ,w_{i}x_{i}\left[N\right]\right]\in C^{N_{t}\times N}$ contains *N* independent unit-power data steams. The simplified model for the total received signal at the BS can thus be expressed as

(11) $$Y_{s}=\alpha H_{s}S+N_{s},$$

where $N_{s}\in C^{N_{t}\times N}$ is the noise matrix, with each column representing the noise vector at a given time instant. This model is crucial for estimating the reflection coefficient α and the channel matrix $H_{s}$, which are essential for target detection and characterization. The reflection coefficient α and the channel $H_{s}$ are used to estimate the target’s location, motion, and other properties.

## 3. Problem Formulation

In this section, we formulate the optimization problem for the integrated system, which includes communication, wireless power transfer, and target sensing. The goal is to find an optimal beamforming strategy that maximizes the system’s overall performance while satisfying constraints on power usage and ensuring the effective operation of all system components. In practice, perfect CSI is probably impossible to obtain. Therefore, it is essential to consider the case of imperfect channel estimation. For simplify notation, we define the actual CSI as $\hat{h}=h+\Delta h$ represents the channel estimation error, satisfying $\Delta h∼N\left(0,\sigma^{2}\right)$ that $\sigma^{2}$ is the variance of Δh. The corresponding received signal at the *k*-th device is then described by

(12) $${\hat{y}}_{k}\left(t\right)=\sqrt{\rho_{k}}\left[h_{k}^{H}s_{k}\left(t\right)+\sum_{j}\Delta h_{k}^{H}s_{j}\left(t\right)+\sum_{j\neq k}h_{k}^{H}s_{j}\left(t\right)+n_{k}\left(t\right)\right]+{\bar{n}}_{k}\left(t\right),$$

where $\Delta h_{k}$ is the reconstructed channel error of the *k*-th device. The achievable data rate for the *k*-th device, which is based on the Shannon capacity formula [41], can be expressed as a function of the SINR as follows:

(13) $${\hat{R}}_{k}=\log_{2}\left(1+\frac{\rho_{k}{\left|h_{k}^{H}w_{k}\right|}^{2}}{\rho_{k}\sum_{j}{\left|\Delta h_{k}^{H}w_{j}\right|}^{2}+\rho_{k}\sum_{j\neq k}{\left|h_{k}^{H}w_{j}\right|}^{2}+\rho_{k}\sigma_{k}^{2}+{\bar{\sigma_{k}}}^{2}}\right),$$

where ${\hat{R}}_{k}$ is the data rate for the *k*-th device. The closed-form upper bound for this rate is derived in Appendix A.

Power transmission in ISCPT systems relies on the ability to transmit energy efficiently to devices, based on the estimated CSI. The power efficiency of energy transfer is related to the power allocated to each device, the channel gain, and the beamforming vector used. As with communication, the beamforming mismatch due to the imperfect CSI will lead to a decrease in the efficiency of power transfer. Thus, the total energy received with imperfect CSI becomes

(14) $$\begin{array}{cc} {\hat{E}}_{k}=\eta_{k}\left(1-\rho_{k}\right) & \left[\underset{\mathrm{Signal}\;\mathrm{Power}\;\mathrm{Term}}{\underset{︸}{\left({\left|h_{k}^{H}w_{k}\right|}^{2}+2ℜ{\left(h_{k}^{H}w_{k}\right)}^{H}\Delta h_{k}+{\left|\Delta h_{k}^{H}w_{k}\right|}^{2}\right)}}\right.\\ \; & \left.+\underset{\mathrm{Interference}\;\mathrm{Power}\;\mathrm{Term}}{\underset{︸}{\sum_{j\neq k}^{K}\left({\left|h_{k}^{H}w_{j}\right|}^{2}+2ℜ{\left(h_{k}^{H}w_{j}\right)}^{H}\Delta h_{k}+{\left|\Delta h_{k}^{H}w_{j}\right|}^{2}\right)}}+\sigma_{k}^{2}\right]. \end{array}$$

For target sensing, we employ the Cramér–Rao bound (CRB) as a fundamental lower bound on the variance of any unbiased estimator. The CRB quantifies the best achievable accuracy for estimating a parameter, such as the target’s position, given a set of measurements [42]. As mentioned above, the parameters of interest are the reflection coefficient α and the channel vector $H_{s}$, which depend on the DOA $\varphi_{DoA}\left(\theta =\varphi_{DoA}\right)$ of the signal from the target. We extend the problem by representing the reflection coefficient α as a tuple of its real and imaginary parts, i.e., $\left\{ℜ\left(\alpha \right),ℑ\left(\alpha \right)\right\}=\left[\alpha_{r},\alpha_{i}\right]$. This allows us to derive the Fisher information matrix (FIM) for the parameters $\tau ={\left[\alpha_{r},\alpha_{i},\theta \right]}^{T}$, which can be written as

(15) $$F=\left[\begin{array}{ccc} F_{\alpha_{r}\alpha_{r}} & F_{\alpha_{r}\alpha_{i}} & F_{\alpha_{r}\theta }\\ F_{\alpha_{i}\alpha_{r}} & F_{\alpha_{i}\alpha_{i}} & F_{\alpha_{i}\theta }\\ F_{\theta \alpha_{r}} & F_{\theta \alpha_{i}} & F_{\theta \theta }. \end{array}\right]$$

According to Formula (11), the likelihood function for the received signal vector $y_{s}$, given the unknown parameters α and θ, is given by the probability density function (PDF) of the received signal, assuming that the noise is complex Gaussian:

(16) $$L\left(\alpha \right)=-\frac{1}{\sigma^{2}}\mathrm{Tr}\left[{\left(Y_{s}-\alpha H_{s}S\right)}^{H}\left(Y_{s}-\alpha H_{s}S\right)\right]+\;\mathrm{constant}\;.$$

The log-likelihood function is a quadratic form in α, so we can differentiate it with respect to τ to find the Fisher information.

The derivative of the log-likelihood function with respect to τ can be expressed as

(17) $$\begin{array}{cc} \; & \frac{\partial L}{\partial \alpha_{r}}=\frac{2}{\sigma^{2}}ℜ\left[S^{H}H_{s}^{H}\left(Y_{s}-\alpha H_{s}S\right)\right], \end{array}$$

(18) $$\begin{array}{cc} \; & \frac{\partial L}{\partial \alpha_{i}}=\frac{2}{\sigma^{2}}ℑ\left[S^{H}H_{s}^{H}\left(Y_{s}-\alpha H_{s}S\right)\right], \end{array}$$

(19) $$\begin{array}{cc} \; & \frac{\partial L}{\partial \theta }=\frac{2}{\sigma^{2}}ℜ\left[S^{H}H_{s}^{H}\left(\frac{\partial \left(\alpha H_{s}S\right)}{\partial \theta }\right)\right]. \end{array}$$

The final expression can use the chain rule of derivative with respect to θ as follows:

(20) $$\frac{\partial \left(\alpha H_{s}S\right)}{\partial \theta }=\left(\frac{\partial \alpha }{\partial \theta }\right)H_{s}S=\left|\alpha \right|\left[-\sin\left(\theta \right)H_{s}S+\cos\left(\theta \right)H_{s}S\right].$$

The Fisher information matrix for the parameters $\alpha_{r}$, $\alpha_{i}$, and θ is the expectation of the second derivatives of the log-likelihood function. Since the noise $n_{s}$ is Gaussian, the Fisher information for each parameter is given by

(21) $$F\left(\alpha \right)=-E\left[\frac{\partial^{2}L}{\partial \alpha^{2}}\right]=\mathrm{CRB}{\left(\alpha \right)}^{-1}.$$

The CRB for the parameters $\alpha_{r}$, $\alpha_{i}$, and θ is the inverse of the Fisher information matrix. For the imperfect CSI case, the calculation of the concrete Fisher information matrix is provided in Appendix B. The CRB for θ can be expressed as

(22) $${\mathrm{CRB}}_{\theta }={\left[F_{\theta \theta }-\frac{F_{\theta \alpha_{r}}F_{\alpha_{r}\theta }}{F_{\alpha_{r}\alpha_{r}}}-\frac{F_{\theta \alpha_{i}}F_{\alpha_{i}\theta }}{F_{\alpha_{i}\alpha_{i}}}\right]}^{-1}.$$

Now, we can formulate the complete optimization problem as follows:

(23a) $$\begin{array}{cc} \underset{w_{k},\Delta h_{k}}{\mathrm{maximize}} & \frac{1}{K}\sum_{k=1}^{K}{\hat{R}}_{k}, \end{array}$$

(23b) $$\begin{array}{cc} \;\mathrm{subject}\;\mathrm{to}\; & {∥w_{k}∥}^{2}\leq P_{\max}\;\forall k,, \end{array}$$

(23c) $$\begin{array}{cc} \; & {\mathrm{SINR}}_{k}\geq \gamma_{k}^{\min}\;\forall k, \end{array}$$

(23d) $$\begin{array}{cc} \; & E_{k}\geq E_{k}^{\min}\;\forall k, \end{array}$$

(23e) $$\begin{array}{cc} \; & {\mathrm{CRB}}_{\theta }\leq \epsilon . \end{array}$$

This optimization problem is a non-convex optimization problem because of the logarithmic terms in the objective function and the quadratic power and energy constraints. Implementing robust beamforming in systems with imperfect CSI is particularly challenging. It is vital to combine multiple objectives that ensure reliable environmental sensing performance under varying interference and environmental conditions, while also maintaining high data rates, low latency for diverse applications, and efficient wireless power transfer with minimal energy overheads. These challenges are further compounded by dynamic real-world conditions, such as user mobility and fluctuating interference levels. Traditional static optimization methods often fall short in addressing these complexities, leading to suboptimal system performance. To tackle these issues, we propose a structured approach that formulates the problem as an adaptive reinforcement learning task and leverages deep learning models to handle the system’s complexity [43].

## 4. Proposed DRL Framework for ISCPT System

In this section, we present a novel DRL framework tailored for optimizing beamforming strategies in the ISCPT system. The main goal is to maximize the information transmission rate while respecting multiple system constraints, such as the maximum transmit power, QoS, and energy-harvesting requirements. DRL is particularly well suited for this task due to its ability to learn complex, adaptive policies in environments where both the state and action spaces are large and continuous. DRL has shown promising results in addressing optimization problems with dynamic and uncertain environments, making it an ideal candidate for wireless communication systems with imperfect CSI. Traditional optimization methods, although effective in simpler, static systems, struggle with the real-time adaptability required in complex and fluctuating environments. The DRL framework, however, allows the learning agent to continually update its policy based on the evolving system state, making it highly adaptable to changing channel conditions [27].

The objective of our optimization, as defined in the objective function (23), is to find an optimal set of beamforming vectors $w_{k}$ that maximize the total information transmission rate of the system, while ensuring the satisfaction of various operational constraints. These constraints are particularly challenging to incorporate into traditional optimization methods but can be effectively handled within the DRL framework. Given the dynamic nature of the ISCPT system, where the beamforming decisions must adapt continuously over time, we propose the use of the deep deterministic policy gradient (DDPG) algorithm to optimize the beamforming vectors in a way that maximizes the overall system performance.

**Figure 2:**
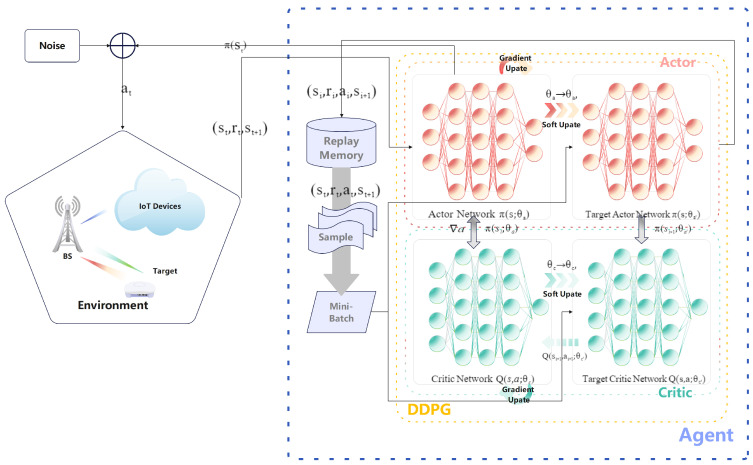
The framework of DRL for the ISCPT system.

As shown in Figure 2, the DRL framework operates in a continuous interaction loop between the agent and the environment. The BS acts as the learning agent, continuously adjusting its beamforming strategy in response to the feedback from the wireless channel. The state information, which includes the channel conditions, power levels, and interference, is collected at each time step. Using this information, the agent learns to select the optimal beamforming vectors that maximize the data rate while respecting system constraints such as SINR, energy-harvesting requirements, and other performance metrics. The DDPG algorithm, which is designed to operate in high-dimensional continuous action spaces, is employed to update the agent’s policy through a combination of actor–critic networks [44].

### 4.1. Action and State Space Definition

To facilitate the learning process, it is crucial to properly define the action and state spaces in the context of the DRL framework. The action space $a_{t}$ refers to the beamforming vectors $w_{k}$ that the agent chooses at each time step. Since the beamforming vectors are complex-valued and high-dimensional, they form a continuous action space. At each time step *t*, the action vector is defined as

(24) $$a_{t}=\left[w_{t,1},w_{t,2},\cdots ,w_{t,K},\right].$$

The state space $s_{t}$, on the other hand, captures all the necessary information about the environment that the agent uses to make its decisions. In the ISCPT system, the state includes information about the channel conditions, such as the channel gains ${\hat{h}}_{t,k}$ for each device, as well as the previous beamforming actions $w_{t-1,k}$ taken by the agent. This information allows the agent to learn the relationship between past actions and their impact on the system performance. The state vector can be expressed as

(25) $$s_{t}=\left[\underset{\mathrm{the}\;\mathrm{current}\;\mathrm{state}\;s^{\prime }}{\underset{︸}{{\hat{h}}_{t,1},{\hat{h}}_{t,2},\ldots ,{\hat{h}}_{t,K},{\hat{h}}_{t,s}}},\underset{\mathrm{the}\;\mathrm{last}\;\mathrm{action}\;a_{t-1}\;}{\underset{︸}{w_{t-1,1},w_{t-1,2},\ldots ,w_{t-1,K}}}\right].$$

The state space is a Markov process, meaning that the future state only depends on the current state and the action taken. This property allows the DRL agent to make decisions based on the current conditions and adapt to changes in the wireless channel over time.

### 4.2. Reward Function Design

The design of the reward function is critical for guiding the learning process and ensuring that the agent maximizes the desired system objective. In our case, the objective is to maximize the average achievable rate while adhering to various operational constraints. The reward function is closely tied to the system’s sum rate, as well as penalties for constraint violations. The reward at each time step is designed to be a weighted combination of the achievable rate and penalties. The total reward is the sum of the achievable rates for all devices:

(26) $$r_{t}=\sum_{k=1}^{K}R_{k}=\sum_{k=1}^{K}\log_{2}\left(1+{\mathrm{SINR}}_{k}\right).$$

In addition to maximizing the sum rate in (23b), the reward function includes penalty terms for violating constraints. For example, the SINR for each device should exceed a minimum threshold $\gamma_{k}$, the energy harvested by the devices should be above a minimum value $E_{\min}$, and the CRB for DoA estimation should remain within a predefined limit ϵ. The reward function can thus be formulated as

(27) $$\begin{array}{cc} \sum_{k=1}^{K}R_{k}=\underset{w_{k},\Delta h_{k}}{\mathrm{maximize}} & \sum_{k=1}^{K}{\hat{R}}_{k}-\lambda_{1}\sum_{k=1}^{K}\left({\mathrm{SINR}}_{k}-\gamma_{k}\right)\\ \; & -\lambda_{2}\sum_{k=1}^{K}\left(E_{k}-E_{\min}\right)-\lambda_{3}\left({\mathrm{CRB}}_{\theta }-\epsilon \right), \end{array}$$

where $\lambda_{1},\lambda_{2},\lambda_{3}$ are positive penalty parameters that balance the trade-off between maximizing the rate and satisfying the constraints. These penalty terms help ensure that the optimization respects the necessary system requirements, such as QoS, energy constraints, and estimation accuracy.

### 4.3. Actor-Critic Networks

The DRL framework employs two neural networks: the actor and the critic. The actor network $\pi \left(s;\theta_{a}\right)$ outputs a deterministic action (beamforming vector) given the current state $s_{t}$, while the critic network $Q\left(s,a;\theta_{c}\right)$ evaluates the quality of the action chosen by the actor. The critic estimates the expected cumulative reward (Q-value) for taking action $a_{t}$ in state $s_{t}$, based on the Bellman equation [45]. The goal of training the actor is to maximize the Q-value, which corresponds to maximizing the average rate (reward). The critic helps to evaluate how good a particular beamforming action is [46].

The objective of the critic network is to minimize the Bellman error, which is the difference between the current Q-value and the target Q-value. The Bellman error is defined as

(28) $$L\left(\theta^{Q}\right)=E\left[{\left(Q\left(s_{t},a_{t}|\theta^{Q}\right)-\left(r_{t}+\gamma Q^{\prime }\left(s_{t+1},a_{t+1}|\theta^{Q^{\prime }}\right)\right)\right)}^{2}\right],$$

where $Q^{\prime }$ is the target critic network, and γ is the discount factor that represents the importance of future rewards.

The actor network is trained to maximize the Q-value output by the critic, i.e., to select actions (beamforming vectors) that maximize the expected cumulative reward. The update rule for the actor is given by

(29) $$\nabla_{\theta^{\pi }}J\left(\theta^{\pi }\right)=E\left[\nabla_{a_{t}}Q\left(s_{t},a_{t}|\theta^{Q}\right)\nabla_{\theta^{\pi }}\pi \left(s_{t}|\theta^{\pi }\right)\right],$$

where $J\left(\theta^{\pi }\right)$ is the objective function for the actor.

To stabilize training, both the actor and critic networks are updated using soft target updates. The target networks $\pi \left(s;\theta_{a^{\prime }}\right)$ and $Q\left(s,a;\theta_{c^{\prime }}\right)$ are updated periodically:

(30) $$\begin{array}{cc} \; & \theta^{\pi^{\prime }}\leftarrow \delta \theta^{\pi }+\left(1-\delta \right)\theta^{\pi^{\prime }},\\ \; & \theta^{Q^{\prime }}\leftarrow \delta \theta^{Q}+\left(1-\delta \right)\theta^{Q^{\prime }}. \end{array}$$

where δ is a small constant that controls the rate of target network updates. The agent stores its experiences $\left(s_{t},a_{t},r_{t},s_{t+1}\right)$ in a replay buffer D. In the procedure of training, the agent samples mini-batches M from the buffer to break the correlation between consecutive experiences. At each time step, the actor network $\pi \left(s;\theta_{a}\right)$ is used to select an action $a_{t}$. To encourage exploration, Ornstein–Uhlenbeck noise is added to the action, making the action selection stochastic [47].

### 4.4. Constraint Handling with Neural Networks

As mentioned earlier, neural networks can be employed to satisfy the constraint conditions in (23b). This is a clever approach that utilizes the transformation of Euler’s formula to meet the constraint of maintaining constant beam values, while also addressing the requirement for complex variables [48]. Only half the number of neurons is required to represent the corresponding beamforming vector. To satisfy the maximum power constraint $P_{m}ax$, a softmax activation function is added, which allows for free adjustment within the given limitations. The corresponding formula can be expressed as follows:

(31) $$w_{k}=\left(\frac{e^{\mu_{2}^{⊤}a_{k}}}{\sum_{i=1}^{K}\mu_{2}^{⊤}a_{i}}\right)\times e^{j\cdot 2\pi \mu_{1}^{⊤}a_{k}},$$

where $\mu_{1}$ and $\mu_{2}$ represent the results of the neuronal output before Euler’s formula and softmax’s activation function, respectively. This method enforces constant beam values while maintaining flexibility for adjusting the beamforming vectors within the required power and phase bounds.

### 4.5. DDPG Algorithm for ISCPT System Optimization

The DDPG algorithm is a model-free, off-policy reinforcement learning approach designed for continuous action spaces, making it ideal for our problem. The algorithm employs two neural networks: the actor network $\pi \left(s;\theta_{a}\right)$, which produces deterministic actions (beamforming vectors), and the critic network $Q\left(s,a;\theta_{c}\right)$, which estimates the expected return for a given state-action pair. The pseudo-code for the DDPG algorithm in the context of ISCPT system optimization is as follows (Algorithm 1):
**Algorithm 1** DDPG algorithm for ISCPT system optimization.1:Initialize actor network $\pi (s;\theta_{a})$ and critic network $Q(s,a;\theta_{c})$ with random parameters.2:Initialize target networks $\pi (s;\theta_{a^{\prime }})$ and $Q(s,a;\theta_{c^{\prime }})$ as copies of the actor and critic networks, respectively.3:Initialize replay buffer D.4:Set the exploration noise parameters Ornstein–Uhlenbeck noise).5:**for** each episode **do**6:    Initialize the state $s_{0}$ (e.g., environment configuration at time step 0).7:    **for** each time step *t* in the episode **do**8:  Select action $a_{t}=\pi \left(s_{t}\right)+N_{t}$ (exploration with noise).9:  Execute action $a_{t}$ in the environment to obtain next state $s_{t+1}$ and reward $r_{t}$.10:  Store the transition $\left(s_{t},a_{t},r_{t},s_{t+1}\right)$ in the replay buffer D.11:  Sample a mini-batch M of transitions $\left(s_{t},a_{t},r_{t},s_{t+1}\right)$ from D.12:  Update critic network by minimizing the loss in (28).13:  Update actor network by maximizing the policy gradient in (29).14:  Update target networks with soft updates in (30).15:    **end for**16:**end for**

This process allows the agent to gradually improve its beamforming strategy through exploration and exploitation. The learning process is repeated across multiple episodes, where each episode represents a new set of channel conditions and environmental configurations. Through iterative training, the agent learns an optimal policy that maximizes the total transmission rate while respecting system constraints.

It is worth noting that the proposed DRL framework demonstrates remarkable robustness, particularly in mitigating the effects of CSI errors, such as $\Delta h_{k}$. Through the combination of online learning, adaptive policy updates, experience replay, multi-task learning, and robust reward function design, the agent is able to continuously adjust its beamforming strategy to accommodate imperfect CSI. This ensures that the system maintains optimal performance even in the presence of channel estimation inaccuracies. Furthermore, the use of neural network regularization and data augmentation techniques enhances the robustness of the model, enabling it to generalize effectively to various real-world scenarios. As a result, the DRL-based approach offers a scalable and reliable solution to the complex optimization problem of robust beamforming in ISCPT systems, overcoming the challenges posed by CSI imperfections.

## 5. Simulation Results

In this section, we present the simulation results for the DRL-based ISCPT system. The results include a detailed analysis of the system’s performance under varying conditions, with particular emphasis on the effects of learning rates, transmit power, SINR, energy harvesting, and CRB on the system’s efficiency and robustness. The parameters of the channel model used in the simulations are summarized in Table 1. We assume a flat-fading channel with AWGN and consider typical parameters for a wireless communication system operating in the C-band at 4 GHz. The IoT devices are randomly deployed within a 50 m radius of the BS, covering a sensing target, and the poss loss is configured as −20 dB with the reference distance 1 m.

In the DRL model for ISCPT systems, several key neural network parameters need to be optimized. The model is trained and tested on a system equipped with a CPU (AMD Ryzen 5 5600X) and a GPU (NVIDIA RTX 3070). These parameters control the training process of the neural network, which aims to optimize beamforming strategies while accounting for power and spectrum constraints. Critical hyperparameters, such as the learning rate, discount factor, batch size, and network architecture, play a crucial role in determining the network’s convergence and performance in multi-objective optimization. These parameters are summarized in Table 2.

A dataset consisting of 1000 samples is used to train the DDPG agent, with 100 samples reserved for validation. The training set is utilized to optimize the beamforming strategy while simultaneously addressing constraints related to power allocation and spectrum management. The validation set is employed to assess the generalization performance of the trained model, ensuring its robustness on statistical data. Both the actor and critic networks are implemented as three-layer deep neural networks (DNNs) to model the continuous control policy and value function, respectively, within the DDPG framework. The actor network is designed to output the beamforming vector $w_{k}$ for each device, given a specific state. It consists of three fully connected layers, each comprising 512 neurons. The ReLU activation function is applied to all layers to introduce non-linearity, enabling the network to approximate complex mappings from state to action effectively. Similarly, the critic network evaluates the quality of the actions selected by the actor by estimating the Q-value. The critic shares the same architecture as the actor network, comprising three fully connected layers with 512 neurons per layer and ReLU activations. This structure enhances the learning capacity and flexibility of the model, facilitating effective training in complex environments.

**Figure 3:**
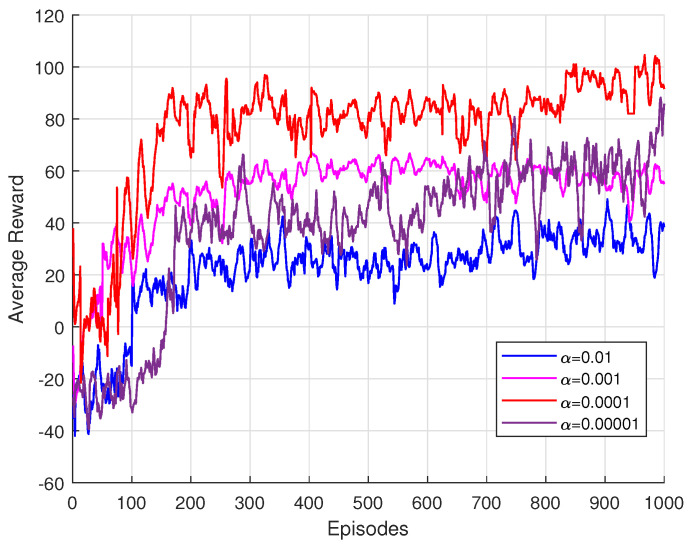
Training performance with different learning rate.

Figure 3 illustrates the learning performance of the proposed DRL-based framework under different learning rates, specifically 0.01, 0.001, 0.0001, and 0.00001. At the start of training, the model receives negative rewards, reflecting its initial difficulty in adapting to the environment’s dynamics. During the first 100 episodes, the system encounters a penalty term in (27), causing the rewards to oscillate within the negative range. This negative oscillation underscores the challenges the agent faces in exploring the solution space effectively during the early training phase. As the model gradually adapts to the environment and learns the underlying channel conditions, the rewards steadily improve, transitioning into a positive incentive phase. The results demonstrate that the learning rate plays a critical role in influencing the model’s training performance. Among the tested values, the learning rate of 0.0001 achieves the best performance, striking an optimal balance between convergence speed and training stability. In contrast, excessively low learning rates impede the model’s ability to generalize, resulting in underfitting and suboptimal performance.

**Figure 4:**
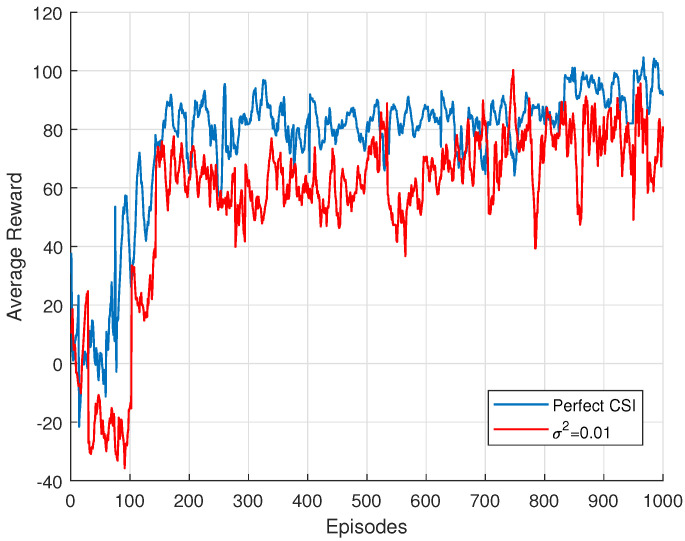
Training performance with different CSI.

As shown in Figure 4, the system with perfect CSI demonstrates faster and more stable convergence, with the rewards steadily increasing and eventually stabilizing at a higher value. In comparison, the system with imperfect CSI experiences a slight degradation in performance. Specifically, the imperfect CSI condition results in slower learning, as evidenced by the delayed rise in rewards. The oscillations are more pronounced, and the overall learning process takes longer to reach the positive reward phase compared to the perfect CSI scenario. This suggests that the uncertainty introduced by imperfect CSI compromises the model’s ability to robustly adapt to the environment, thereby slowing down the learning process and affecting performance stability.

**Figure 5:**
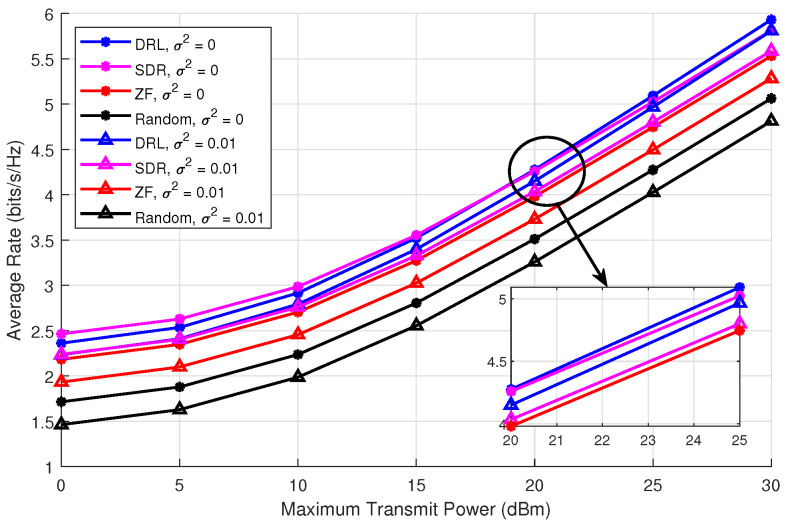
Average rate vs. transmit power under *K* = 4, $E_{k}^{\min}$ = 0 dBm, ϵ = 0.1.

Next, we evaluate the average rate of the ISCPT system as a function of the maximum transmit power, which ranges from 0 dBm to 30 dBm. The results, presented in Figure 5, demonstrate how the average rate varies with transmit power. Here, DRL refers to the method proposed in this paper, while semidefinite relaxation (SDR) represents a optimal solution as explored in previous studies [21]. ZF indicates beamforming via the zero-forcing algorithm, and random denotes random beamforming.

The experimental results demonstrate that as the transmit power increases, the average rate of all algorithms improves due to enhanced signal strength and resource utilization. Among the algorithms tested, the DRL-based approach consistently outperforms SDR, ZF, and Random under both perfect $\left(\sigma^{2}=0\right)$ and imperfect $\left(\sigma^{2}=0.01\right)$ CSI conditions, and it provides a good stability compared to other algorithms even with the channel error. DRL exhibits exceptional robustness, maintaining high performance in the presence of CSI imperfections, which significantly degrade the performance of other algorithms. While SDR performs well under perfect CSI, it suffers from a substantial performance decline with imperfect CSI due to its reliance on precise channel information. ZF offers moderate performance but is limited by its sensitivity to channel estimation errors. Random beamforming predictably performs the worst, highlighting the necessity of optimization strategies.

**Figure 6:**
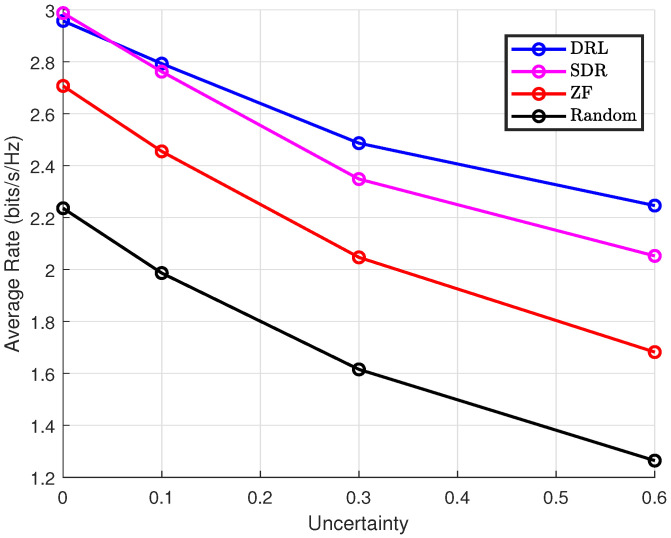
Average rate vs. CSI uncertainty under *K* = 4, $P_{max}$ = 10 dBm, $E_{k}^{\min}$ = 0 dBm, ϵ = 0.1.

In practical systems, it is crucial to adapt to various external environmental changes, including delays, frequency offsets, and inherent errors in channel estimation, which make it difficult to guarantee the accuracy of CSI. Therefore, we need to adopt a method that is both adaptive and stable. As shown in Figure 6, the DRL method demonstrates significant robustness compared to other approaches under varying levels of uncertainty. To further emphasize the robustness of DRL, we analyze the percentage decrease in the average rate as CSI uncertainty increases. Specifically, as the uncertainty rises from 0 to 0.6, the rate reduction for DRL is notably smaller than for SDR, ZF, and Random methods. The results clearly show that the DRL method exhibits superior robustness under increasing uncertainty. At σ=0.6, DRL retains 80% of its original rate, while SDR retains only 64.3%, ZF 58.3%, and Random 48.8%. This significant difference highlights the advantage of DRL in adapting to channel uncertainty and maintaining stable system performance, making it a more reliable solution in scenarios with uncertain or imperfect CSI.

From a computational complexity perspective, DRL, despite its superior performance and robustness, has a higher computational overhead due to its iterative learning process and the training phase, which involves neural network updates and extensive interactions with the environment. The complexity is given by $O\left(2N_{epi}T_{max}\left(\sum_{l_{=}1}^{L}n_{l-1}\cdot n_{l}+n_{l}\cdot b_{l}\right)\right)$, where *n* and *b* represent the amounts of neurons and bias, respectively. However, once trained, the DRL model operates with relatively low complexity during inference, making it suitable for real-time applications. In contrast, SDR relies on solving semidefinite programming (SDP) problems with a complexity of $O\left(t{\left(N_{t}\left(K+1\right)\right)}^{3.5}\right)$, which makes it computationally prohibitive for large-scale systems. These results underscore the trade-off between computational complexity and performance, with DRL emerging as the most robust and efficient approach for dynamic, real-time scenarios.

**Figure 7:**
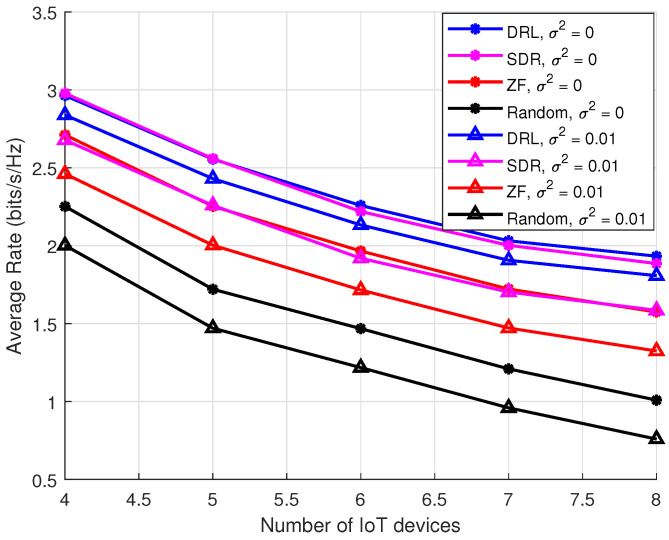
Average rate vs. the number of IoT devices under $P_{max}$ = 10 dBm, $E_{k}^{\min}$ = 0 dBm, ϵ = 0.1.

Further, we compare the average IoT rate for varying numbers of devices. Figure 7 illustrates the influence of different deviations of imperfect CSI on the system’s performance, reflected by the average rate for different numbers of IoT devices. As σ increases, representing greater uncertainty in the CSI, the system’s performance gradually deteriorates. Specifically, higher σ values result in increased instability in the optimization problem, making it more challenging to achieve stable performance. These results highlight the superior robustness and performance of the DRL-based beamforming algorithm under imperfect CSI conditions. By continuously adjusting its policy based on environmental feedback, the DRL model effectively manages uncertainty and adapts to dynamic changes in the wireless channel. In contrast, traditional beamforming algorithms, which rely on static or approximate models, are more prone to performance degradation in high-uncertainty environments.

The results further reinforce the superior robustness and adaptability of the DRL-based beamforming algorithm, particularly in environments where CSI is imperfect or subject to noise. Unlike traditional methods, which rely on static or approximate models and are more sensitive to inaccuracies in CSI, the DRL-based model can continuously adapt to changing conditions, effectively mitigating the effects of imperfect information. This capability positions DRL as a promising solution for next-generation wireless networks, where channel conditions are often dynamic and unpredictable.

**Figure 8:**
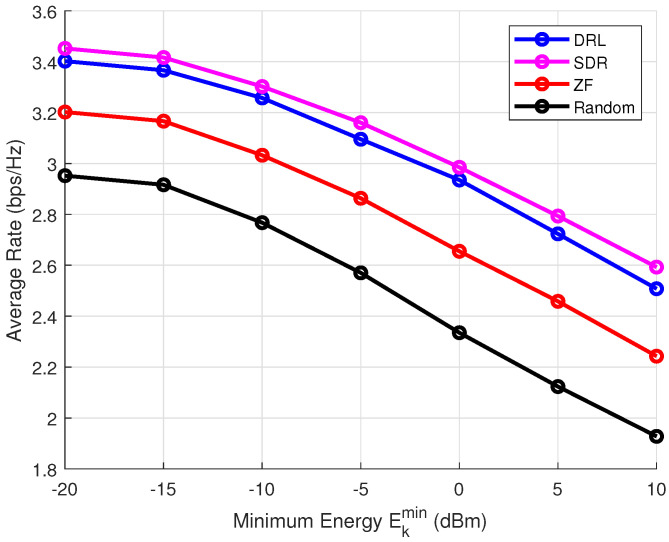
Impact of energy harvesting on transmission rate under $P_{\max}$ = 10 dBm, *K* = 4, ϵ = 0.1, $\sigma^{2}$ = 0.

**Figure 9:**
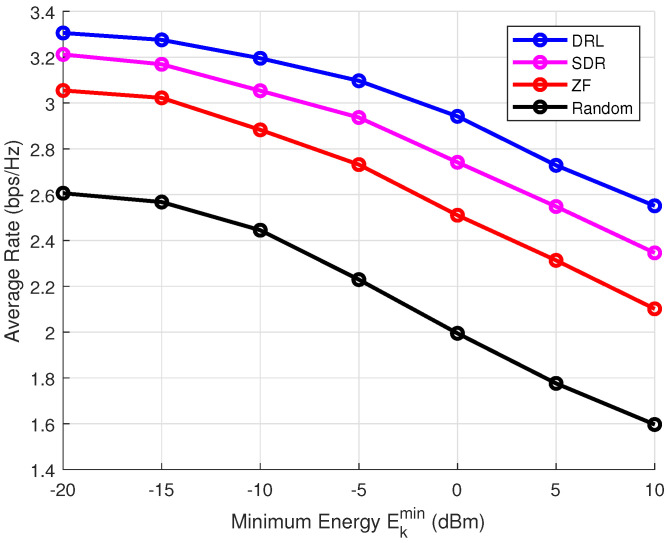
Impact of energy harvesting on transmission rate under $P_{\max}$ = 10 dBm, *K* = 4, ϵ = 0.1, $\sigma^{2}$ = 0.1.

Then, we investigate the impact of the minimum energy-harvesting requirement on the transmission rate. Figure 8 and Figure 9 compare the average rates achieved by different algorithms without and with CSI error. The results clearly show that the DRL-based beamforming algorithm significantly outperforms other methods, particularly in scenarios where energy-harvesting constraints are present. In ISCPT systems, energy fluctuations can directly impact the quality of communication, as the available energy is crucial for powering transmitters, receivers, and related electronics. A decrease in energy availability can result in lower transmission power, leading to signal degradation and performance loss. The DRL-based beamforming approach, by continuously adapting to the available energy, ensures that the system can maintain a high level of performance even in the presence of energy fluctuations.

**Figure 10:**
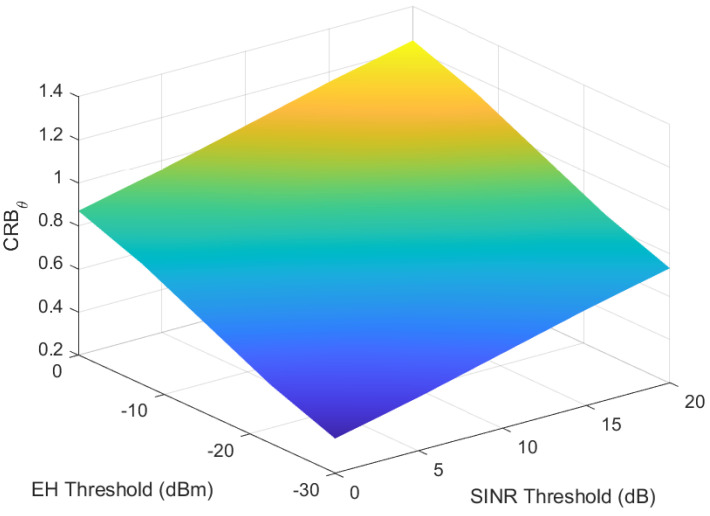
Impact of transmit power and energy on CRB.

Finally, we analyze the influence of transmit power and energy harvesting on the CRB for angle estimation, ${\mathrm{CRB}}_{\theta }$, as shown in Figure 10. Both transmit power and energy harvesting contribute significantly to reducing the CRB, thereby improving the accuracy of angle estimation. This, in turn, enhances the overall system performance by providing more precise angle information for beamforming. The results underscore the critical role of transmit power and energy harvesting in optimizing the performance of the system, particularly in scenarios requiring accurate angle estimation for effective beamforming.

## 6. Conclusions

In this section, we presented the simulation results for a DRL-based ISCPT system. The results demonstrated the effects of various parameters, such as transmit power, energy harvesting, SINR, and CRB, on the system’s performance. The DRL algorithm successfully adapted to these parameters, improving the system’s efficiency and robustness under dynamic conditions. Future work will focus on further optimizing the DRL algorithm and exploring advanced learning techniques to enhance system performance. Additionally, we plan to investigate non-linear energy-harvesting models to better capture real-world energy transfer dynamics. Furthermore, research will be extended to hybrid beamforming techniques, aiming to achieve more efficient and flexible beamforming designs that balance complexity and performance in integrated sensing and communication systems.

## Figures and Tables

**Table 1:** Channel model parameters for ISCPT system.

Symbol	Parameter	Value
*F*	Carrier frequency	4 GHz
$N_{t}$	Transmit antenna	64
$a_{G}$	Path loss exponents	3
κ	Rician factor	2.2
α	Target reflection coefficient	0.01
η	Energy conversion efficiency	0.7
$\sigma^{2}$	Noise Power	−70 dBm

**Table 2:** DDPG algorithm for ISCPT system parameters.

Symbol	Parameter	Value
$N_{epi}$	The number of episodes	1000
$T_{max}$	The number of maxstep in each episode	100
$\alpha_{a}$	Learning rate for actor network	0.0001
$\alpha_{c}$	Learning rate for critic network	0.0001
$\delta_{a}$	Target actor network update rate	0.0001
$\delta_{c}$	Target critic network update rate	0.0001
λ	Decaying rate	0.00001
γ	Discount factor	0.95
M	The size of mini-batch	32
D	Replay buffer size	1,000,000
U	The number of steps’ synchronization	1

## Data Availability

The original contributions presented in this study are included in this article; further inquiries can be directed to the corresponding author.

## Abbreviations

The following abbreviations are used in this manuscript:

ISCPT	Integrated sensing, communication, and power transfer
QoS	Quality of service
DRL	Deep reinforcement learning
DDPG	Deep deterministic policy gradient
CSI	Channel state information
uRLLC	Ultra-reliable low-latency communication
mMTC	Massive machine-type communications
eMBB	Enhanced mobile broadband
ISAC	Integrated sensing and communications
SWIPT	Simultaneous wireless information and power transfer
IoT	Internet of Things
MmWave	Millimeter wave
AI	Artificial intelligence
DL	Deep learning
RIS	Reconfigurable intelligent surface
MAC	Media access control
LOS	Line of sight
NLOS	Non-line of sight
ULA	Uniform linear array
AoD	Angle of departure
AWGN	Additive white Gaussian noise
SINR	Signal-to-interference-plus-noise ratio
DOA	Direction of arrival
ToF	Time of flight
CRB	Cramér–Rao bound
PDF	Probability density function
FIM	Fisher information matrix
DNN	Deep neural network
SDR	Semidefinite relaxation

## Appendix A

To derive an upper bound I on ${\hat{R}}_{k}$, we can define the terms inside the denominator for simplicity:

- $h_{k}^{H}w_{k}=h_{k}$, the desired signal channel gain.
- $h_{k}^{H}w_{j}=h_{kj}$, the interference channel gain.
- $\Delta h_{k}^{H}w_{j}=\Delta h_{kj}$, the error term for the interference channel gain.

Thus, the expression for (13) becomes

(A1) $${\hat{R}}_{k}=\log_{2}\left(1+\frac{\rho_{k}{\left|h_{k}\right|}^{2}}{\rho_{k}\sum_{j}{\left|\Delta h_{kj}\right|}^{2}+\rho_{k}\sum_{j\neq k}{\left|h_{kj}\right|}^{2}+\rho_{k}\sigma_{k}^{2}+{\bar{\sigma }}_{k}^{2}}\right)\leq I.$$

In our case, we can apply the Cauchy–Schwarz inequality to bound the term $\sum_{j}{\left|\Delta h_{kj}\right|}^{2}$. This term is the sum of squared errors in the channel estimates, so applying the Cauchy–Schwarz inequality gives the following:

(A2) $$\sum_{j}{\left|\Delta h_{kj}\right|}^{2}\leq {\left(\sum_{j}\left|\Delta h_{kj}\right|\right)}^{2},$$

and the error term $\sum_{j}{\left|\Delta h_{kj}\right|}^{2}$ can be bounded as follows: $\sum_{j}{\left|\Delta h_{kj}\right|}^{2}\leq N\cdot \max_{j}{\left|\Delta h_{kj}\right|}^{2}$.

For a concave function, like the logarithm, we can apply Jensen’s inequality:

(A3) $$E\left[\log_{2}\left(1+X\right)\right]\leq \log_{2}\left(1+E\left[X\right]\right).$$

Let us define the argument inside the logarithm as *X*:

(A4) $$X=\frac{\rho_{k}{\left|h_{k}\right|}^{2}}{\rho_{k}\sum_{j}{\left|\Delta h_{kj}\right|}^{2}+\rho_{k}\sum_{j\neq k}{\left|h_{kj}\right|}^{2}+\rho_{k}\sigma_{k}^{2}+{\bar{\sigma }}_{k}^{2}}$$

Now, apply Jensen’s inequality to bound ${\hat{R}}_{k}$ that can replace $E\left[{\left|\Delta h_{kj}\right|}^{2}\right]=\sigma_{\Delta }^{2}$:

(A5) $$E\left[{\hat{R}}_{k}\right]\leq \log_{2}\left(1+\frac{\rho_{k}{\left|h_{k}^{H}w_{k}\right|}^{2}}{\rho_{k}\cdot N\cdot \sigma_{\Delta }^{2}+\rho_{k}\sum_{j\neq k}{\left|h_{k}^{H}w_{j}\right|}^{2}+\rho_{k}\sigma_{k}^{2}+{\bar{\sigma_{k}}}^{2}}\right).$$

Therefore, the upper bound for the achievable rate $R_{k}$ is given by

(A6) $${\hat{R}}_{k}\leq R_{k}=\log_{2}\left(1+\frac{\rho_{k}{\left|h_{k}\right|}^{2}}{\rho_{k}\cdot N\cdot \max_{j}{\left|\Delta h_{kj}\right|}^{2}+\rho_{k}\sum_{j\neq k}{\left|h_{kj}\right|}^{2}+\rho_{k}\sigma_{k}^{2}+{\bar{\sigma }}_{k}^{2}}\right)$$

## Appendix B

To derive the CRB for the parameters involving the imperfect CSI ${\hat{H}}_{s}=H_{s}+\Delta H_{s}$, we need to go through the following inference procedure. It is similar to (16), which is the log-likelihood function of L:

(A7) $$L\left(\alpha_{r},\alpha_{i},\theta |{\hat{H}}_{s}\right)=-N\log\left(\pi \sigma^{2}\right)-\frac{1}{\sigma^{2}}{∥Y_{s}-\alpha \left(H_{s}+\Delta H_{s}\right)S∥}_{F}^{2}.$$

The derivative of the log-likelihood function with respect to $\alpha_{r}$ is

(A8) $$\frac{\partial L}{\partial \alpha_{r}}=\frac{2}{\sigma^{2}}ℜ\left[S^{H}H_{s}^{H}\left(Y_{s}-\alpha H_{s}S\right)\right]+\frac{2}{\sigma^{2}}ℜ\left[S^{H}H_{s}^{H}\left(-\alpha \Delta H_{s}S\right)\right],$$

where the second term represents the contribution of the estimation error $\Delta H_{s}$.

Similarly, the derivative of the log-likelihood function with respect to $\alpha_{i}$ is

(A9) $$\frac{\partial L}{\partial \alpha_{i}}=\frac{2}{\sigma^{2}}ℑ\left[S^{H}H_{s}^{H}\left(Y_{s}-\alpha H_{s}S\right)\right]+\frac{2}{\sigma^{2}}ℑ\left[S^{H}H_{s}^{H}\left(-\alpha \Delta H_{s}S\right)\right].$$

The derivative with respect to θ is more complicated because it involves the phase of α. Using the chain rule, we obtain

(A10) $$\frac{\partial L}{\partial \theta }=\frac{2}{\sigma^{2}}ℜ\left[S^{H}H_{s}^{H}\left(-|\alpha |\sin\left(\theta \right)H_{s}S\right)\right]+\Gamma ,$$

where Γ is the terms involving $\Delta H_{s}$. The Fisher information can be computed as follows:

(A11) $$\begin{array}{cc} \; & F_{\alpha_{r}\alpha_{r}}=\frac{2}{\sigma^{2}}E\left[S^{H}H_{s}^{H}H_{s}S\right]+\frac{2}{\sigma^{2}}E\left[S^{H}H_{s}^{H}\Delta H_{s}S\right], \end{array}$$

(A12) $$\begin{array}{cc} \; & F_{\alpha_{i}\alpha_{i}}=\frac{2}{\sigma^{2}}E\left[S^{H}H_{s}^{H}H_{s}S\right]+\frac{2}{\sigma^{2}}E\left[S^{H}H_{s}^{H}\Delta H_{s}S\right], \end{array}$$

(A13) $$\begin{array}{cc} \; & F_{\theta \theta }=\frac{2}{\sigma^{2}}E\left[S^{H}H_{s}^{H}H_{s}S\right]+\frac{2}{\sigma^{2}}E\left[S^{H}H_{s}^{H}\Delta H_{s}S\right], \end{array}$$

(A14) $$\begin{array}{cc} \; & F_{\theta \alpha_{r}}=\frac{2}{\sigma^{2}}E\left[S^{H}H_{s}^{H}\left(-|\alpha |\cos\left(\theta \right)H_{s}S\right)\right], \end{array}$$

(A15) $$\begin{array}{cc} \; & F_{\theta \alpha_{i}}=\frac{2}{\sigma^{2}}E\left[S^{H}H_{s}^{H}\left(-|\alpha |\cos\left(\theta \right)H_{s}S\right)\right]. \end{array}$$

Given the symmetry of FIM, $F_{\alpha_{r}\theta }$ and $F_{\alpha_{i}\theta }$ also have the same expression.
